# Association between ambient temperature and genitourinary emergency ambulance dispatches in Japan: A nationwide case-crossover study

**DOI:** 10.1097/EE9.0000000000000298

**Published:** 2024-02-14

**Authors:** Yasuko Mano, Lei Yuan, Chris Fook Sheng Ng, Masahiro Hashizume

**Affiliations:** a Keck School of Medicine of the University of Southern California, Los Angeles, California; b Department of Global Health Policy, Graduate School of Medicine, The University of Tokyo, Tokyo, Japan

**Keywords:** Climate change, Genitourinary disease, Ambient temperature, Emergency ambulance dispatches, Morbidity

## Abstract

**Background:**

Although the effects of temperature on genitourinary morbidity and mortality have been investigated in several countries, it remains largely unexplored in Japan. We investigated the association between ambient temperature and genitourinary emergency ambulance dispatches (EADs) in Japan and the modifying roles of sex, age, and illness severity.

**Methods:**

We conducted a time-stratified case-crossover study with conditional quasi-Poisson regression to estimate the association between mean temperature and genitourinary EADs in all prefectures of Japan between 2015 and 2019. A mixed-effects meta-analysis was used to pool the association at the country level. Subgroup analyses were performed to explore differences in associations stratified by sex, age, and illness severity.

**Results:**

We found an increased risk of genitourinary EAD associated with higher temperatures. The cumulative relative risk (RR) at the 99th temperature percentile compared with that at the 1st percentile was 1.74 (95% confidence interval (CI) = [1.60, 1.89]). We observed higher heat-related RRs in males (RR = 1.89; 95% CI = [1.73, 2.07]) than females (RR = 1.56; 95% CI = [1.37, 1.76]), and in the younger (RR = 2.13; 95% CI = [1.86, 2.45]) than elderly (RR = 1.39; 95% CI = [1.22, 1.58]). We found a significant association for those with mild or moderate cases (RR = 1.77; 95% CI = [1.62, 1.93]), but not for severe or life-threatening cases (RR = 1.20; 95% CI = [0.80, 1.82]).

**Conclusion:**

Our study revealed heat effects on genitourinary EADs in Japan. Men, youth, and mild-moderate illnesses were particularly vulnerable subgroups. These findings underscore the need for preventative measures aimed at mitigating the impact of temperature on genitourinary emergencies.

**What this study adds:**

Our findings reveal the association between ambient temperature and genitourinary emergency ambulance dispatches at both the prefecture and country levels in Japan, and we examine the modifying effects of sex, age, and illness severity. Our research is the first study to investigate the effects of temperature on genitourinary morbidity using national emergency ambulance dispatch data as the outcome—a sensitive acute health indicator—and is one of the few studies that has conducted subgroup analyses to further identify subpopulations that at higher risks. Furthermore, the study is one of few studies that has examined the impacts of both hot and cold temperatures on kidney diseases, as opposed to most extant studies that have only focused on heat effects.

## Introduction

Climate change is a public health crisis that has been affecting the global population.^1^ Recent studies have shown increased interest in the effects of ambient temperature on morbidity and mortality from various diseases.^2,3^ Most of the studies have reported increased risks of cardiovascular and respiratory diseases associated with nonoptimal temperature.^4–7^ However, much less is known about the impact of daily temperature on other conditions such as kidney diseases despite the annualized rate of change of kidney dysfunction significantly increasing by 0.35% over the years 1990–2019.^8^

Although empirical evidence has indicated possible associations between nonoptimal temperature and kidney diseases, the findings have been inconsistent. Some studies have reported an increased risk of acute kidney injury (AKI),^9–11^ hemodialysis initiation,^12^ or mortality from renal diseases^13–15^ associated with high temperatures, while others have found no such association.^16^ So far, relatively few studies have examined the impacts of both hot and cold temperatures, and most have focused solely on the influence of heat.^1,9,13,15^ Furthermore, previous studies have tended to focus on specific genitourinary conditions, with few investigating all genitourinary conditions simultaneously. Moreover, limited studies have been conducted to further identify particularly susceptible subpopulations that are at higher risk of nonoptimal temperature-related genitourinary conditions. These points emphasize the importance of conducting a comprehensive analysis of nonoptimal temperature-related genitourinary morbidity and mortality. Previous nationwide studies in Japan have examined the associations between daily temperature and renal cause-specific mortality using different temperature indexes and have mainly focused on the relationships between temperature and genitourinary mortality.^17,18^ To our knowledge, our study is the first globally to investigate the effects of daily mean temperature on genitourinary morbidity using national emergency ambulance dispatches (EADs) registries, a sensitive acute health indicator.

In the present study, we investigated the associations between ambient temperature and genitourinary EADs over the full temperature spectrum for the 47 prefectures in Japan from 2015 to 2019. Subgroup analyses were further conducted to examine the modifying effects of sex, age, and disease severity.

## Methods

### Data collection

We used EAD data for all prefectures in Japan from the Fire and Disaster Management Agency of the Ministry of Internal Affairs and Communication.^19^ Daily EAD data for genitourinary causes were collected for the 47 prefectures of Japan from the period of 1 January 2015 to 31 December 2019 (except for Tokyo prefecture; data were available from 1 January 2016 to 31 December 2019). All genitourinary causes of EADs were coded according to the International Classification of Diseases, tenth revision (ICD-10: codes N00-N99). Each genitourinary EAD case recorded information regarding sex, age group (17 years and younger, 18–64 years old, and 65 years and older), and severity of illness.^20^ The illness severity was classified into six categories (mild, moderate, severe, life-threatening, death, or other) and was documented by the physician who performed the initial assessment of the patient upon their arrival at the hospital.^21^

We collected daily mean temperature and relative humidity data for the study period from representative weather stations maintained by the Japan Meteorological Agency in each of the 47 prefectures. Daily mean dew point temperatures were calculated from the mean temperature and relative humidity.^22,23^

### Statistical analysis

We conducted a two-stage meta-analysis to investigate the association between mean temperature and genitourinary EADs at both the country and prefecture levels. All modeling and statistical computations were performed using R statistical software (Version 4.2.1; R Development Core Team) with the R packages *dlnm*,^24^ *mixmeta*.^25^

In the first stage, we used a time-stratified case-crossover study design with a conditional quasi-Poisson regression model to estimate the association between mean temperature and genitourinary EADs in each prefecture. The seasonality and long-term trend were controlled by a time stratum in the model that matched case-control days by selecting the same day of the week within the same month and year.^26^ To describe the nonlinear and delayed associations, the model incorporated a distributed lag nonlinear model using a cross-basis function for temperature and lag.^27^ Specifically, the exposure-response association was modeled with a quadratic B-spline with two internal knots placed at the 33rd and 66th percentiles of the prefecture-specific temperature distribution. The lag–response association was modeled with a natural cubic spline with an intercept and two internal knots equally spaced in the log scale. The lag period was extended to 14 days. The number and placement of knots were determined based on the quasi-Akaike information criterion. We also included a natural cubic spline of a 3-day moving average dew point temperature with three degrees of freedom and a binary variable to control for the public holidays. We adjusted for dew point temperature instead of relative humidity as a time-varying confounding factor in the model because dew point temperature is an absolute measure of humidity, thereby making it a reliable measure unlike relative humidity, which depends strongly on temperature and can therefore cause increased diurnal variation.^28,29^ Statistical analysis results without adjusting for dew point temperature are also generated for comparison.

In the second stage, we applied a mixed-effects meta-analysis to pool the prefecture-specific coefficients obtained from the first stage to obtain the nationwide estimates. We included prefecture-specific mean temperature and temperature range as meta-predictors to address between-prefecture variability that can be explained by differences in temperature distributions. Residual heterogeneity was tested and then quantified by the multivariate extension of the Cochran *Q*-test and *I*-square statistic.^30,31^ This meta-regression model was subsequently utilized to derive the best linear unbiased prediction (BLUP) of the cumulative exposure-response associations across all prefectures. The analytical strategy using BLUP enables prefectures with limited daily genitourinary EAD counts or short series, potentially associated with imprecise estimates, to leverage information from larger populations with similar characteristics.^30^ The prefecture and pooled curves were then scaled by reentering them on the minimum risk temperature percentile (MMTP) of the temperature distribution of each prefecture and at the country level, defined as the minimum of the exposure-response curve. The search for the minimum risk temperature was limited between the 1st–99th percentile range.^32^ We also summarized the results by computing the cold- and heat-related cumulative risk ratios (RRs) at the 1st and 99th percentiles from these curves, using the MMTP as the reference. Country-pooled lag–response relationships for heat and cold were also derived from the re-centered estimates using prefecture-specific MMTPs derived from the BLUPs.^31^

Subgroup analyses were performed stratified by sex, age (younger, <65 years; older, ≥65 years), and two illness severity groups (mild to moderate, and severe to life-threatening) using the same two-staged modeling explained above.^20^

To evaluate the robustness of our findings, we conducted several sensitivity analyses. Initially, we altered the number of knots from two, located at the 33rd and 66th percentiles, to three knots placed at the 25th, 50th, and 75th percentiles and the 50th, 75th, and 90th percentiles for modeling the exposure-response relationship. For the lag–response relationship, we changed the number of knots from two to three equally spaced knots and the lag period from 14 to 7 days. This study is exempt from ethics approval since the analysis was based on anonymized data.

## Results

Table 1 shows the summary statistics for genitourinary EAD at the country level. A total of 639,994 genitourinary EADs were included in the study. The daily mean number of genitourinary EADs was 7.5 (Table 1). Of all genitourinary EADs recorded for the study period, over half of the cases (57.9%) were males, and approximately half of the cases (50.7%) were from the older age category. The majority (96.3%) were from the mild to moderate severity category. Table 2 shows the distribution of prefecture-level meteorological variables. The mean ambient temperature during the study period was 16.0 °C and varied considerably from 9.6 °C to 23.8 °C. The mean dew point at the country level was 10.1 °C and ranged from 3.6 °C to 18.7 °C. (Supplemental Figure 1; http://links.lww.com/EE/A262 displays a distinct and consistent seasonality for total genitourinary cases and daily mean temperature. We found that a higher number of genitourinary cases occurred at high temperatures. The summary statistics of genitourinary causes of EADs and weather variables for each prefecture are shown in Supplemental Tables 1 and 2; http://links.lww.com/EE/A262).

**Table 1.** Descriptive statistics for genitourinary emergency ambulance dispatches (EADs) from 2015 to 2019 in Japan^a^

	All counts (%)	Daily mean (SD)
Overall	639,994	7.5 (10.8)
Sex^b^		
Male	370,722 (57.9)	4.3 (6.1)
Female	267,438 (41.8)	3.1 (5.1)
Age category (year)		
Younger (<65)	315,823 (49.3)	3.7 (6.0)
Older (≥65)	324,171 (50.7)	3.8 (5.2)
Severity category^c^		
Mild or moderate	616,011 (96.3)	7.2 (10.7)
Severe or worse	23,734 (3.7)	0.3 (0.6)

a For Tokyo, EAD data were available from 2016 to 2019.

b There were 1,098 (0.29%) cases missing sex information.

c There were 249 (0.04%) cases categorized as other types of severity.

SD indicates standard deviation.

**Table 2.** Summary statistics for meteorological variables from 2015 to 2019 at country level

Meteorological variables	Mean	SD	Minimum	25th	Median	75th	Maximum
Mean temperature (°C)^a^	16.0	2.3	9.6	15.2	16.4	17.3	23.8
Dew point (°C)^a^	10.1	2.3	3.6	9.2	10.2	11.2	18.7

25th and 75th are percentiles.

a The summary statistics were calculated based on the prefecture-level averages of each meteorological variable.

SD indicates standard deviation.

**Figure 1.**
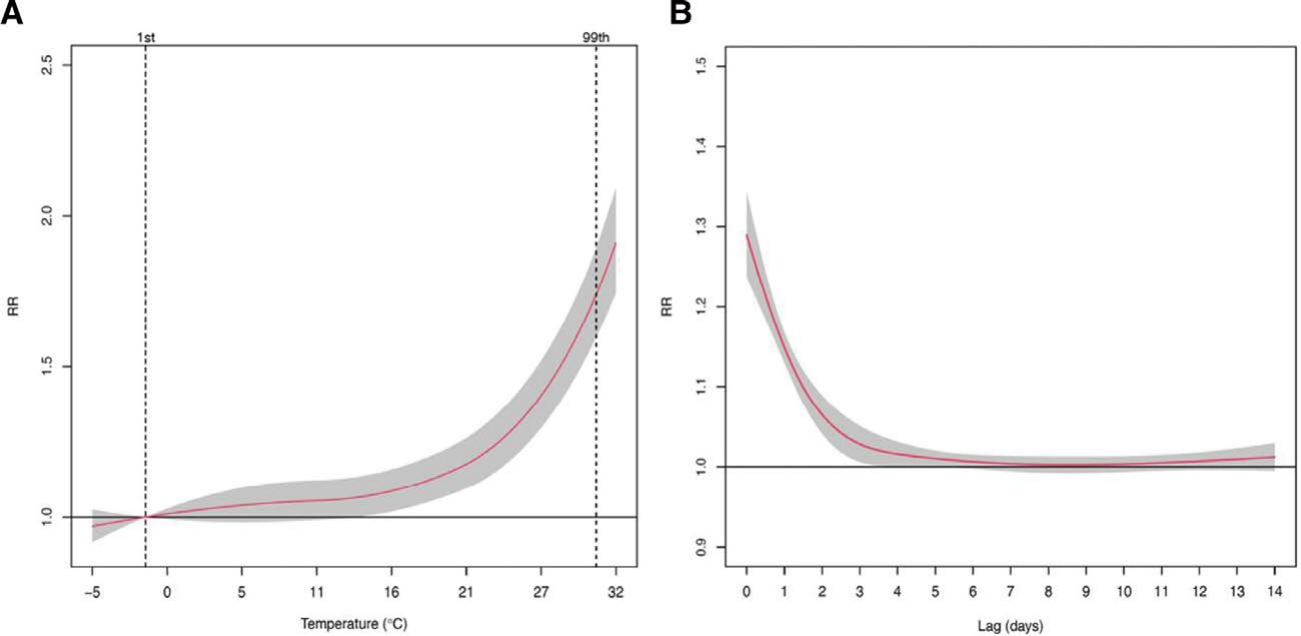
A, Exposure-response curve with 95% empirical CI (shaded gray) and (B) Lag–response associations for emergency ambulance dispatch (EAD) over the extended lag period of up to 14 days with 95% CI (shaded gray) in Japan, estimated by using a conditional Poisson model adjusting for seasonality, long-term time trend, the day of week, holiday, and dew point. Vertical lines indicate the location for the minimum risk temperature (1st) and RR calculations for cold (1st) or heat (99th). CI indicates confidence interval; RR, relative risk.

Figure 1 shows the pooled exposure-response curve between mean temperature and genitourinary cause of EADs. Results from the analysis of heterogeneity are illustrated in Supplemental Table 3; http://links.lww.com/EE/A262, with a comparison of statistics from the simple multivariate random-effects meta-analysis and meta-regressions with a single or both meta-predictors. The pooled estimates from the multivariate meta-regression model indicated low heterogeneity at the country level (*I*-square statistic = 10.0%; multivariate Cochran *Q*-test for residual heterogeneity: *Q* = 195.5195, *P* = 0.15). The association between temperature and all genitourinary causes of EADs was nonlinear, and the minimum risk temperature was identified at the 1st percentile of temperature. Heat-related risks of genitourinary causes of EADs increased as mean temperature increased. A significant heat risk was observed with a RR of 1.74 (95% confidence interval [CI] = 1.60, 1.89) at the 99th percentile compared with the MMTP (Table 3). The pooled lag–response curve for all genitourinary causes of EADs at the country level showed a delayed effect for heat and lasted for up to 5 days (Supplemental Figure 2; http://links.lww.com/EE/A262).

**Table 3.** Pooled RRs of genitourinary emergency ambulance dispatch (EAD) in Japan

	Temperature percentile^a^	RRs (95% CI)	
	MMTP^b^ (MMT)	Cold risk^c^	Heat risk^c^
Overall	1 (−1.4)	1.00	1.74 (1.60, 1.89)
Sex			
Male	9 (4.1)	1.01 (0.95, 1.08)	1.89 (1.73, 2.07)
Female	1 (−1.4)	1.00	1.56 (1.37, 1.76)
Age category (year)			
Younger (<65)	1 (−1.4)	1.00	2.13 (1.86, 2.45)
Older (≥65)	1 (−1.4)	1.00	1.39 (1.22, 1.58)
Severity category			
Mild or moderate	1 (−1.4)	1.00	1.77 (1.62, 1.93)
Severe or life-threatening	1 (−1.4)	1.00	1.20 (0.80, 1.82)

Pooled cumulative RRs with 95% CIs for genitourinary EADs for overall and subpopulations, estimated by using a conditional Poisson model adjusting for seasonality, long-term time trend, day of week, holiday, and dew point.

a The percentiles of the temperature for centering and RR calculation.

b The percentile of minimum risk temperature, identified between the 1st and 99th percentiles of temperature at the country level.

c The cold and heat risks are the RRs at the 1st (cold) and 99th (heat) percentiles of temperature.

CI indicates confidence interval; MMTP, minimum risk temperature percentile; RR, risk ratio.

**Figure 2.**
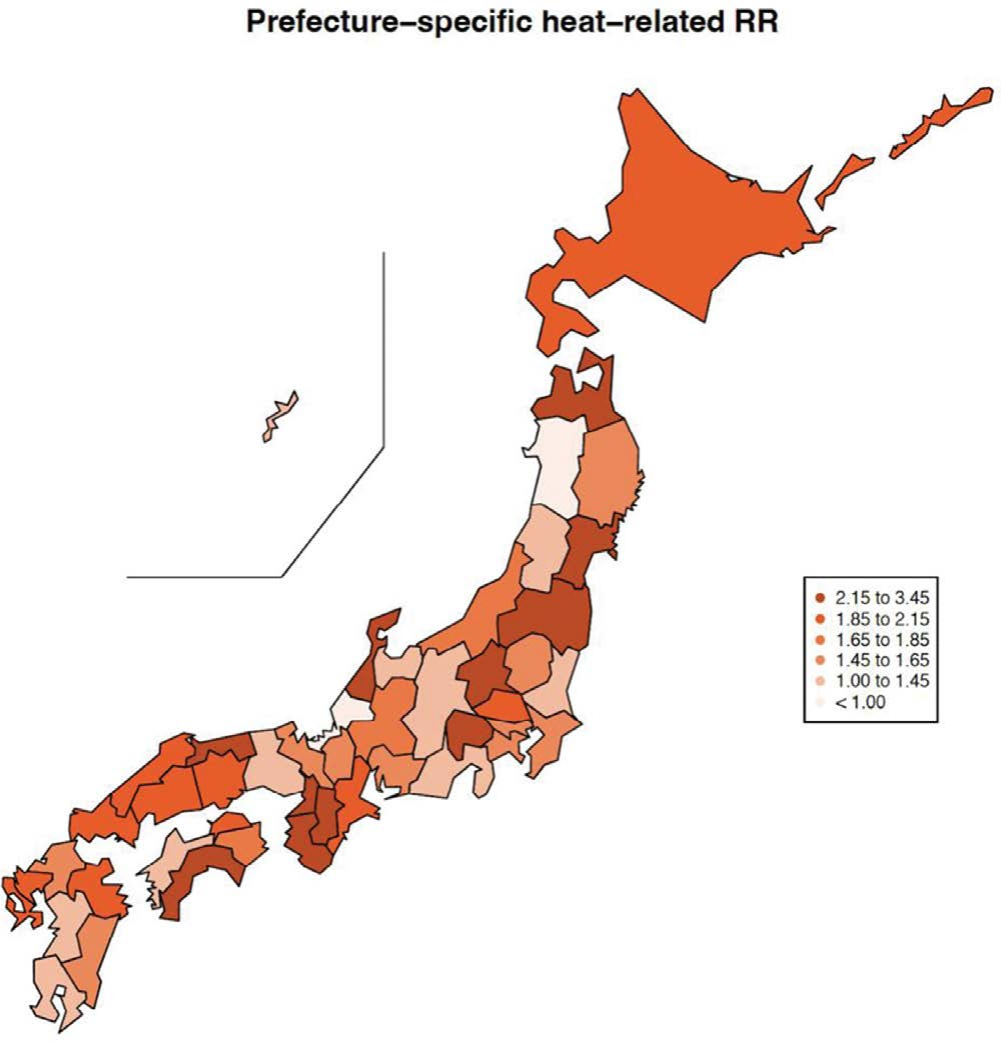
Spatial map of prefecture-specific relative risks (RRs) of heat for all genitourinary emergency ambulance dispatch (EAD). MMT(P)s varied by prefecture, and the locations for RR calculations were the 99th percentiles of each prefecture’s mean temperature. See Table S3; http://links.lww.com/EE/A262 for the corresponding numeric data.

Similar trends were also seen for prefecture-specific heat-related RRs of genitourinary EADs (Figure 2 and Supplemental Table 4; http://links.lww.com/EE/A262). Figure 2 shows the spatial distribution of RRs for heat in the 47 prefectures in Japan. In general, heat-related RRs tended to vary across prefectures. Some of the highest heat-related RRs of genitourinary EADs were seen in Miyagi, with a RR of 2.6 (95% CI = 1.3, 5.0), Fukushima, with a RR of 2.8 (95% CI = 1.5, 5.1), and Wakayama, with a RR of 3.0 (95% CI = 1.5, 6.0). The lowest RR was seen in Akita prefecture, with a RR of 0.9 (95% CI = 0.4, 2.2). The prefecture-specific RRs for heat are summarized in (Supplemental Table 4; http://links.lww.com/EE/A262).

**Figure 3.**
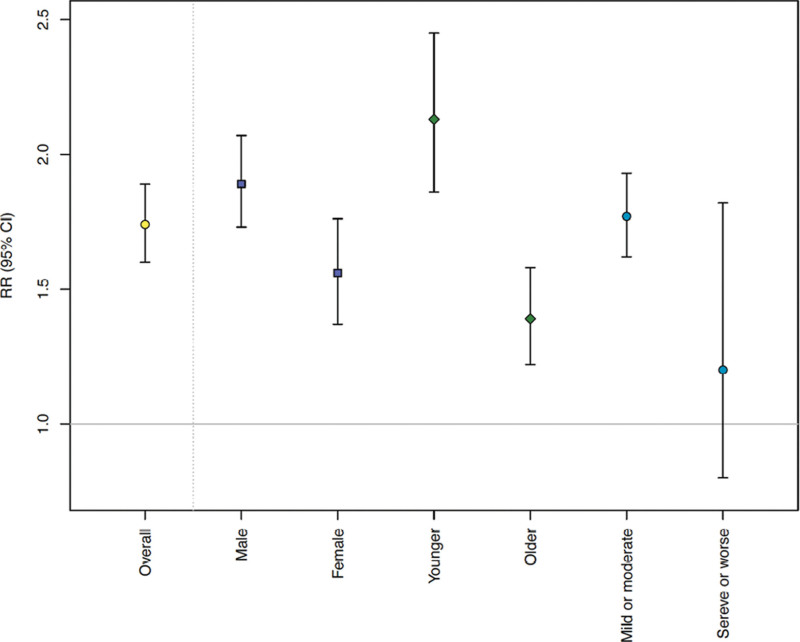
Pooled cumulative RRs with 95% CIs (vertical bars) for heat-related genitourinary emergency ambulance dispatch (EAD) in subgroup analyses by sex, age (younger, <65 years of age; older, ≥65 years of age), and severity groups, estimated by using a conditional Poisson model adjusting for seasonality, long-term time trend, the day of week, holiday, and dew point. The heat risks are the RRs at the 99th percentile of temperature, compared with the respective minimum risk temperature percentile. CI indicates confidence interval; RR, relative risk.

Figure 3 shows the overall cumulative RRs in the subgroup analyses stratified by sex, age, and illness severity groups. Consistent MMTPs are identified for the general population and all subpopulations, at the 1st temperature percentile (Table 3). The RRs of all genitourinary causes of EADs for heat differed among all the subgroups, and CIs were generally not overlapping. Specifically, the heat-related RRs of all genitourinary causes of EADs were higher in males (RR = 1.89; 95% CI = 1.73, 2.07) and younger people (RR = 2.13; 95% CI = 1.86, 2.45) than in females (RR = 1.56; 95% CI = 1.37, 1.76) and older people (RR = 1.39; 95% CI = 1.22, 1.58), respectively. Furthermore, we found a significant association for those with mild or moderate cases (RR = 1.77; 95% CI = 1.62, 1.93), but not for those with severe or life-threatening cases (RR = 1.20; 95% CI = 0.80, 1.82).

A slight difference for heat-related RRs was observed in models with and without adjusting for dew point temperature, although with consistent patterns (Supplemental Table 5; http://links.lww.com/EE/A262). Sensitivity analyses showed that the estimates were robust against alternative specification for the knots of temperature and lag period (Supplemental Table 6; http://links.lww.com/EE/A262). The prefecture-specific exposure-response associations obtained in the first stage (without BLUP) were also analyzed, and the association estimates and exposure-response curves changed only slightly (Supplemental Figure 3; http://links.lww.com/EE/A262).

## Discussion

While there have been previous studies that have investigated the associations of ambient temperature with numerous health outcomes such as hospital admission rates, hemodialysis initiation, and mortality from AKI or chronic kidney disease (CKD) to our knowledge, there have been no studies that have examined the associations using EADs. The goal of this study was to assess the associations between ambient temperature and EADs due to genitourinary causes in Japan. Additionally, the study investigated these associations among different subgroup populations based on sex, age, and illness severity. Overall, we found that there are heat-related effects on EADs due to genitourinary conditions at both the country level and the prefecture-level. In addition, we found evidence of risk increase associated with heat exposure among the male, younger (<65 years old) patients, and those with mild or moderate cases of genitourinary conditions.

Our results regarding the heat-related effects on RRs of genitourinary conditions are consistent with a previous study that concluded that higher ambient temperatures were associated with increased risk of kidney stone hospital admissions in the United States, a study that showed that higher temperatures were associated with end-stage renal disease (ESRD) incidence in Okinawa, Japan, and a study that found that an increase in the number of hospitalizations from CKD overlapped with the occurrence of heatwaves.^33–35^ However, our results differ from a study in Thailand that found no associations between heatwaves and renal failure mortality^36^ and from studies that have shown that the risk of hemodialysis initiation or mortality due to ESRD was higher in the cold, winter seasons.^37,38^ Additionally, our results are inconsistent with another study that concluded that winter was an independent risk factor for all-cause in-hospital mortality for AKI^39^ and a recent time-series study that looked at 204 countries and found that the overall CKD death burden associated with lower temperatures was higher than that associated with warmer temperatures.^40^

Our subgroup analysis results have some consistencies and inconsistencies with previous studies as well—for example, our findings that men and younger populations are at a higher risk of genitourinary conditions were not quite the same as a previous study in Queensland, Australia that concluded that men and elderly are more vulnerable to heat effects causing genitourinary diseases.^41^ While the reason behind this difference remains unclear, it may suggest that the elderly population in Japan has better protection against heat, whether it be through preventative healthcare measures, genetics, or living environments; alternatively, this difference may indicate that the younger population in Japan has a different mobility pattern compared with that in Australia that puts them at an increased risk of exposure. Furthermore, our results differ from another study in South Korea that found that cold spells are associated with hospital admission and mortality due to AKI, especially in the elderly population.^42^ However, our findings share similar findings with another study in Taiwan that concluded that the risk of hospitalization due to CKD in warmer temperatures was higher in the male population.^43^

While the exact mechanism for the relationship between ambient temperature and genitourinary EADs is unknown, a possible explanation is that heat in general adds stress to the kidneys due to causes such as dehydration. In hot temperatures, significant stress is added to the renal system, particularly because dehydration leads to lowered blood pressure, which would in turn lead to decreased kidney function and increase chances of kidney injury/damage.^1,44^ This explanation would make sense in the setting of our findings not revealing statistically significant cold effects.

To our knowledge, our study is the first to investigate the association between ambient temperature and genitourinary EADs in Japan. One of the strengths of the current study is the use of emergency data, a sensitive indicator that better reflects the acute and nonfatal effects of nonoptimal temperatures compared with other health indicators such as mortality and hospitalization. Another strength of the study is the 5-year time scale and well-covered spatial resolution for all 47 prefectures, allowing for a large sample size and thus the strong statistical power to examine the association at both prefecture and country levels. Third, the current study investigated the entire temperature spectrum within the context, including both heat and cold effects. Moreover, the application of the time-stratified case-crossover design with a distributed lag nonlinear model allows us to assess the short-term and delayed impact of temperature on genitourinary morbidity precisely. In the meantime, our results demonstrated the need to pay additional attention to adjustment for other time-varying confounders such as absolute humidity when estimating heat-related effects, as the time-stratified case-crossover design might not fully capture the time variations in the humidity exposure. This contrasts with most studies that have focused solely on either heat or cold. Last, we also conducted subgroup analyses to further identify susceptible subpopulations and the potential role of age, sex, and illness severity in explaining the differences.

Some limitations of our study include the fact that our study only looked at the 47 prefectures of Japan, therefore the results may not be generalizable to the populations of other countries. Second, there are potential misclassifications of the meteorological and EAD data that could bias the estimates given that the exposure level was measured from representative weather stations in cities, and the primary diagnosis of each EAD case was recorded with a single ICD code. Although the initial assessments performed by the physicians are standardized across Japan, there remains possibility of over- or underestimation of temperature-related EAD risks given that the diagnoses and disease severities were determined from a preliminary exam by the first responders, rather than them being a definitive diagnosis. Additionally, due to the low proportion of severe and worse genitourinary conditions of EAD (3.7%), our results should be interpreted with caution when comparing with other studies assessing more fatal conditions. Further research on specific disease categories is also necessary to properly understand how heat affects genitourinary health. Moreover, our current research investigated all genitourinary conditions grouped together and did not conduct any subgroup analyses based on subtypes of genitourinary conditions; future studies may benefit from such subgroup analyses, but further data collection would be needed to obtain a sufficient sample size. Finally, our study only conducted subgroup analyses based on sex, age, and severity category, but future studies could benefit from the inclusion of other variables, such as race—however, given that the ethnic composition of the population of Japan is 99% Japanese and 0.6% other,^45^ race was not included in our current study.

In conclusion, our study found that the risk of genitourinary EADs increases with higher temperatures, with particularly notable effects on the young, male population who have mild to moderate genitourinary cases. These findings highlight the importance of heat adaptation strategies in preventing the occurrence of genitourinary EADs, especially in vulnerable populations.

## Acknowledgments

We would like to acknowledge the Fire and Disaster Management Agency (FDMA) of the Ministry of Internal Affairs and Communication in Japan, and the Japan Meteorological Agency (JMA) for providing the data. The interpretation and reporting of these data are the responsibility of the authors and in no way should be seen as an official policy of interpretation of FDMA or the JMA.

## Conflicts of interest statement

The authors declare that they have no conflicts of interest with regard to the content of this report.

## Funding

This project was supported by the Japan Science and Technology Agency (JST) as part of SICORP, Grant Number JPMJSC20E4, and the Environment Research and Technology Development Fund (JPMEERF23S21120) of the Environmental Restoration and Conservation Agency provided by Ministry of the Environment of Japan. This work was also supported by JST SPRING, Grant Number JPMJSP2108 (recipient: L.Y.).

## Supplementary material

Supplemental digital content is available through direct URL citations in the HTML and PDF versions of this article (www.environepidem.com).

## Supplementary Material



## References

[1] Johnson RJ Sánchez-Lozada LG Newman LS. Climate change and the kidney. Ann Nutr Metab. 2019;74:38–44. 31203298 10.1159/000500344

[2] Gasparrini A Guo Y Hashizume M. Mortality risk attributable to high and low ambient temperature: a multicountry observational study. Lancet. 2015;386:369–375. 26003380 10.1016/S0140-6736(14)62114-0 PMC4521077

[3] Wu Y Li S Zhao Q. Global, regional, and national burden of mortality associated with short-term temperature variability from 2000–19: a three-stage modelling study. Lancet Planet Health. 2022;6:e410–e421. 35550080 10.1016/S2542-5196(22)00073-0 PMC9177161

[4] Song X Wang S Hu Y. Impact of ambient temperature on morbidity and mortality: an overview of reviews. Sci Total Environ. 2017;586:241–254. 28187945 10.1016/j.scitotenv.2017.01.212

[5] Tong M Wondmagegn B Xiang J. Hospitalization costs of respiratory diseases attributable to temperature in Australia and projections for future costs in the 2030s and 2050s under climate change. Int J Environ Res Public Health. 2022;19:9706. 35955062 10.3390/ijerph19159706 PMC9368165

[6] Lei J Chen R Yin P. Association between cold spells and mortality risk and burden: a nationwide study in China. Environ Health Perspect. 2022;130:27006. 35157500 10.1289/EHP9284 PMC8843087

[7] Ryti NRI Mäkikyrö EMS Antikainen H. Cold spells and ischaemic sudden cardiac death: effect modification by prior diagnosis of ischaemic heart disease and cardioprotective medication. Sci Rep. 2017;7:41060. 28106161 10.1038/srep41060 PMC5247694

[8] Murray CJL Aravkin AY Zheng P. Global burden of 87 risk factors in 204 countries and territories, 1990–2019: a systematic analysis for the global burden of disease study 2019. Lancet. 2020;396:1223–1249. 33069327 10.1016/S0140-6736(20)30752-2 PMC7566194

[9] Chapman CL Johnson BD Parker MD Hostler D Pryor RR Schlader Z. Kidney physiology and pathophysiology during heat stress and the modification by exercise, dehydration, heat acclimation and aging. Temperature (Austin). 2021;8:108–159. 33997113 10.1080/23328940.2020.1826841 PMC8098077

[10] Lim YH So R Lee C. Ambient temperature and hospital admissions for acute kidney injury: a time-series analysis. Sci Total Environ. 2018;616-617:1134–1138. 29089137 10.1016/j.scitotenv.2017.10.207

[11] Borg M Bi P Nitschke M Williams S McDonald S. The impact of daily temperature on renal disease incidence: an ecological study. Environ Health. 2017;16:114. 29078794 10.1186/s12940-017-0331-4 PMC5659014

[12] Ogata S Yorioka N. Environmental factors influencing the survival of chronic dialysis patients. Clin Exp Nephrol. 2011;15:405–409. 21249416 10.1007/s10157-010-0400-2

[13] Gasparrini A Armstrong B Kovats S Wilkinson P. The effect of high temperatures on cause-specific mortality in England and Wales. Occup Environ Med. 2012;69:56–61. 21389012 10.1136/oem.2010.059782

[14] Stafoggia M Forastiere F Agostini D. Factors affecting in-hospital heat-related mortality: a multi-city case-crossover analysis. J Epidemiol Community Health. 2008;62:209–215. 18272735 10.1136/jech.2007.060715

[15] Remigio RV Jiang C Raimann J. Association of extreme heat events with hospital admission or mortality among patients with end-stage renal disease. JAMA Netw Open. 2019;2:e198904–e198904. 31397862 10.1001/jamanetworkopen.2019.8904 PMC6692691

[16] Liu J Hansen A Varghese B. Cause-specific mortality attributable to cold and hot ambient temperatures in Hong Kong: a time-series study, 2006–2016. Sustain Cities Soc. 2020;57:102131.

[17] Lee W Kim Y Honda Y Kim H. Association between diurnal temperature range and mortality modified by temperature in Japan, 1972–2015: investigation of spatial and temporal patterns for 12 cause-specific deaths. Environ Int. 2018;119:379–387. 30005186 10.1016/j.envint.2018.06.020

[18] Ma C Yang J Nakayama SF Honda Y. The association between temperature variability and cause-specific mortality: evidence from 47 Japanese prefectures during 1972–2015. Environ Int. 2019;127:125–133. 30913457 10.1016/j.envint.2019.03.025

[19] Fire and Disaster Management Agency. Fire and Disaster Management Agency. Published 2020. Available at: https://www.fdma.go.jp/. Accessed 16 March 2023.

[20] Kubo R Ueda K Seposo X Honda A Takano H. Association between ambient temperature and intentional injuries: a case-crossover analysis using ambulance transport records in Japan. Sci Total Environ. 2021;774:145511. 33609821 10.1016/j.scitotenv.2021.145511

[21] Kotani K Ueda K Seposo X. Effects of high ambient temperature on ambulance dispatches in different age groups in Fukuoka, Japan. Glob Health Action. 2018;11:1437882. 29471745 10.1080/16549716.2018.1437882 PMC5827789

[22] Davis RE McGregor GR Enfield KB. Humidity: a review and primer on atmospheric moisture and human health. Environ Res. 2016;144:106–116. 26599589 10.1016/j.envres.2015.10.014

[23] Lawrence MG. The relationship between relative humidity and the dewpoint temperature in moist air: a simple conversion and applications. Bull Am Meteorol Soc. 2005;86:225–234.

[24] Gasparrini A. Distributed lag linear and non-linear models in R: the package dlnm. J Stat Softw. 2011;43:1–20. PMC3191524 22003319

[25] Sera F Armstrong B Blangiardo M Gasparrini A. An extended mixed-effects framework for meta-analysis. Stat Med. 2019;38:5429–5444. 31647135 10.1002/sim.8362

[26] Armstrong BG Gasparrini A Tobias A. Conditional Poisson models: a flexible alternative to conditional logistic case cross-over analysis. BMC Med Res Methodol. 2014;14:122. 25417555 10.1186/1471-2288-14-122 PMC4280686

[27] Gasparrini A. Modeling exposure-lag-response associations with distributed lag non-linear models. Stat Med. 2014;33:881–899. 24027094 10.1002/sim.5963 PMC4098103

[28] Armstrong B Sera F Vicedo-Cabrera AM. The role of humidity in associations of high temperature with mortality: a multicountry, multicity study. Environ Health Perspect. 2019;127:97007. 31553655 10.1289/EHP5430 PMC6792461

[29] Baldwin JW Benmarhnia T Ebi KL Jay O Lutsko NJ Vanos JK. Humidity’s role in heat-related health outcomes: a heated debate. Environ Health Perspect. 2023;131:55001. 37255302 10.1289/EHP11807 PMC10231239

[30] Gasparrini A Armstrong B Kenward MG. Multivariate meta-analysis for non-linear and other multi-parameter associations. Stat Med. 2012;31:3821–3839. 22807043 10.1002/sim.5471 PMC3546395

[31] Gasparrini A Guo Y Hashizume M. Temporal variation in heat–mortality associations: a multicountry study. Environ Health Perspect. 2015;123:1200–1207. 25933359 10.1289/ehp.1409070 PMC4629745

[32] Tobías A Hashizume M Honda Y. Geographical variations of the minimum mortality temperature at a global scale: a multicountry study. Environ Epidemiol. 2021;5:e169. 34934890 10.1097/EE9.0000000000000169 PMC8683148

[33] Zhao Q Li S Coelho MSZS. The association between heatwaves and risk of hospitalization in Brazil: a nationwide time series study between 2000 and 2015. PLoS Med. 2019;16:e1002753. 30794537 10.1371/journal.pmed.1002753 PMC6386221

[34] Gronlund CJ Zanobetti A Schwartz JD Wellenius GA O’Neill MS. Heat, heat waves, and hospital admissions among the elderly in the United States, 1992-2006. Environ Health Perspect. 2014;122:1187–1192. 24905551 10.1289/ehp.1206132 PMC4216145

[35] Kaufman J Vicedo-Cabrera AM Tam V Song L Coffel E Tasian G. The impact of heat on kidney stone presentations in South Carolina under two climate change scenarios. Sci Rep. 2022;12:369. 35013464 10.1038/s41598-021-04251-2 PMC8748744

[36] Huang C Cheng J Phung D Tawatsupa B Hu W Xu Z. Mortality burden attributable to heatwaves in Thailand: a systematic assessment incorporating evidence-based lag structure. Environ Int. 2018;121:41–50. 30172927 10.1016/j.envint.2018.08.058

[37] Iseki K Morita O Fukiyama K. Seasonal variation in the incidence of end-stage renal disease. Am J Nephrol. 1996;16:375–381. 8886173 10.1159/000169028

[38] Goto S Hamano T Ogata S Masakane I. Seasonal variations in cause-specific mortality and transition to renal replacement therapy among patients with end-stage renal disease. Sci Rep. 2020;10:2325. 32047207 10.1038/s41598-020-59153-6 PMC7012814

[39] Li J Zhou Q Zhang D Wang J Yang L. Seasonal variation in the detection rate and all-cause in-hospital mortality of AKI in China: a nationwide cohort study. Front Public Health. 2022;10:947185. 36262238 10.3389/fpubh.2022.947185 PMC9575196

[40] He L Xue B Wang B. Impact of high, low, and non-optimum temperatures on chronic kidney disease in a changing climate, 1990–2019: a global analysis. Environ Res. 2022;212:113172. 35346653 10.1016/j.envres.2022.113172 PMC9907637

[41] Lu P Xia G Zhao Q. Attributable risks of hospitalizations for urologic diseases due to heat exposure in Queensland, Australia, 1995-2016. Int J Epidemiol. 2022;51:144–154. 34508576 10.1093/ije/dyab189 PMC8855997

[42] Kim KN Shin MK Lim YH. Associations of cold exposure with hospital admission and mortality due to acute kidney injury: a nationwide time-series study in Korea. Sci Total Environ. 2023;863:160960. 36528107 10.1016/j.scitotenv.2022.160960

[43] Zafirah Y Lin YK Andhikaputra G Sung FC Deng LW Wang YC. Mortality and morbidity of chronic kidney disease associated with ambient environment in metropolitans in Taiwan. Atmos Environ. 2022;289:119317. 10.1371/journal.pone.0253814 PMC8259956 34228742

[44] Sasai F Roncal-Jimenez C Rogers K. Climate change and nephrology. Nephrol Dial Transplant. 2023;38:41–48. 34473287 10.1093/ndt/gfab258 PMC9869860

[45] Hisashige A. History of healthcare technology assessment in Japan. Int J Technol Assess Health Care. 2009;25:210–218. 19527539 10.1017/S0266462309090655

