# Supplementary material for: Association between ambient temperature and genitourinary emergency ambulance dispatches in Japan: A nationwide case-crossover study
Source: Environ Epidemiol. 2024 Feb 14;8(2):e298. doi: 10.1097/EE9.0000000000000298 (PMC11008653; doi:10.1097/EE9.0000000000000298)
Supplement: Supplementary file 1 [file ee9-8-e298-s001.docx]

**Supplemental Materials**

**Association between ambient temperature and genitourinary emergency ambulance dispatches in Japan: A nationwide case-crossover study**

**Yasuko Mano^1*^, Lei Yuan^2*^, Chris Fook Sheng Ng^2^, Masahiro Hashizume^2^**

^1^Keck School of Medicine of the University of Southern California, Los Angeles, CA, United States of America

^2^Department of Global Health Policy, Graduate School of Medicine, The University of Tokyo, Tokyo, Japan

^*^Co-first authors

Address correspondence to Masahiro Hashizume, Department of Global Health Policy, Graduate School of Medicine, The University of Tokyo, -3-1 Hongo, Bunkyo-ku, Tokyo 113-0033, Japan. Telephone: (81) 3 5841 3688. Email: hashizume@m.u-tokyo.ac.jp

**Supplemental Material Table of Contents**

**Supplemental Figure 1.** Time-series plots for mean temperature (blue) and genitourinary emergency ambulance dispatches (EADs, red) in representative prefectures of Japan from 2015-2019.

**Supplemental Figure 2.** Lag-response curve for heat effect on genitourinary emergency ambulance dispatches (EADs) at the country level in Japan for (A) overall population, as well as by (B) sex, (C) age and (D) disease severity sub-groups, with 95% empirical confidence interval (shaded).

**Supplemental Figure 3.** Exposure–response associations with and without best linear unbiased prediction (with 95% empirical CI, shaded grey) in representative prefectures of Japan.

**Supplemental Table 1.** Descriptive statistics for genitourinary emergency ambulance dispatches (EADs) from 2015 to 2019 in Japan.

**Supplemental Table 2.** Summary statistics for meteorological variables from 2015 to 2019 at the prefecture level.

**Supplemental Table 3.** Multivariate Wald test on significance of each meta-predictor in explaining variation in overall cumulative temperature-mortality association in the second stage mixed-effects meta-regression models.

**Supplemental Table 4.** Prefecture-specific association between temperature and genitourinary emergency ambulance dispatches (EADs) in Japan among the overall population.

**Supplemental Table 5.** Pooled RRs of genitourinary emergency ambulance dispatch (EAD) in Japan without adjustment for dew point.

**Supplemental Table 6.** Alternative statistical modelling for the pooled cumulative and lag associations between temperature and genitourinary emergency ambulance dispatch (EAD) at the country level.

| **Supplemental Figure 1.** Time-series plots for mean temperature (blue) and genitourinary emergency ambulance dispatches (EADs, red) in representative prefectures of Japan from 2015-2019. For Tokyo, EAD data were available from 2016 to 2019.  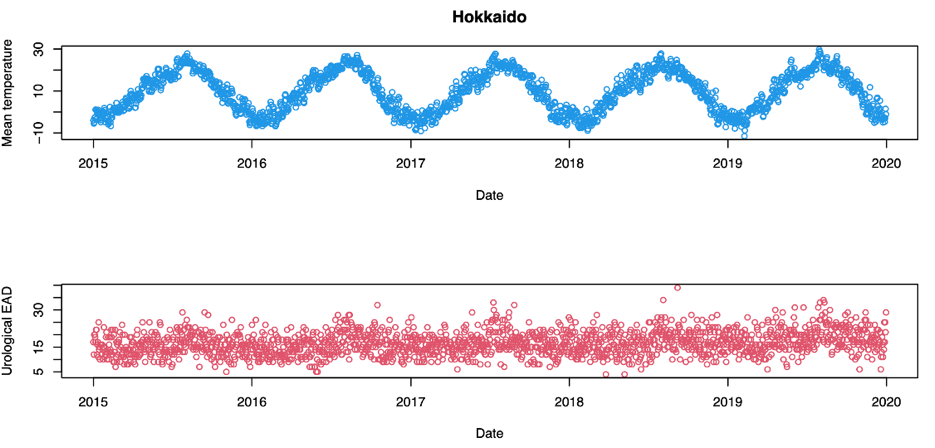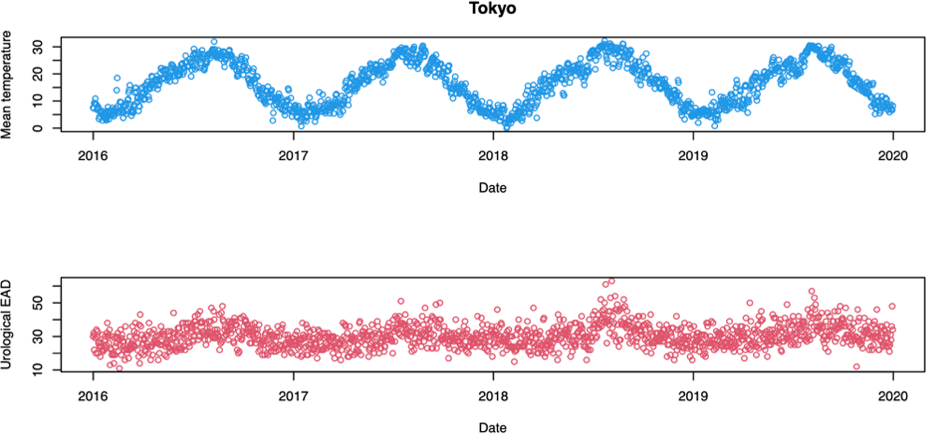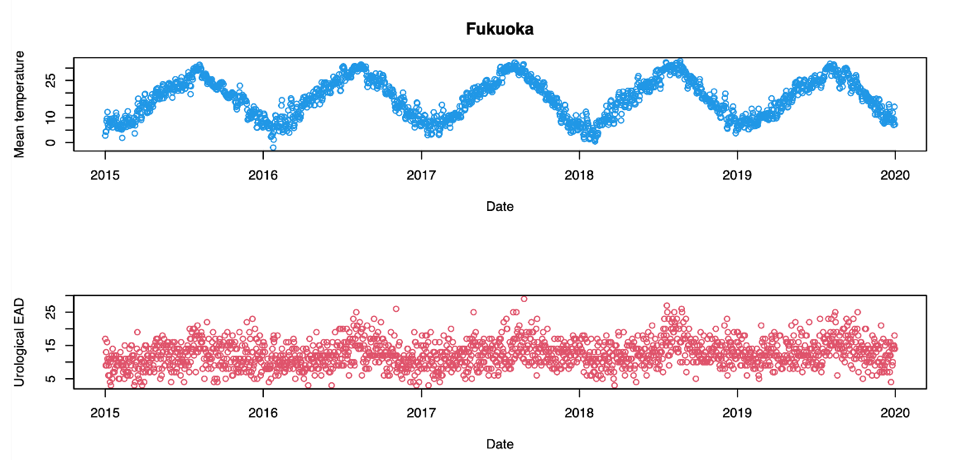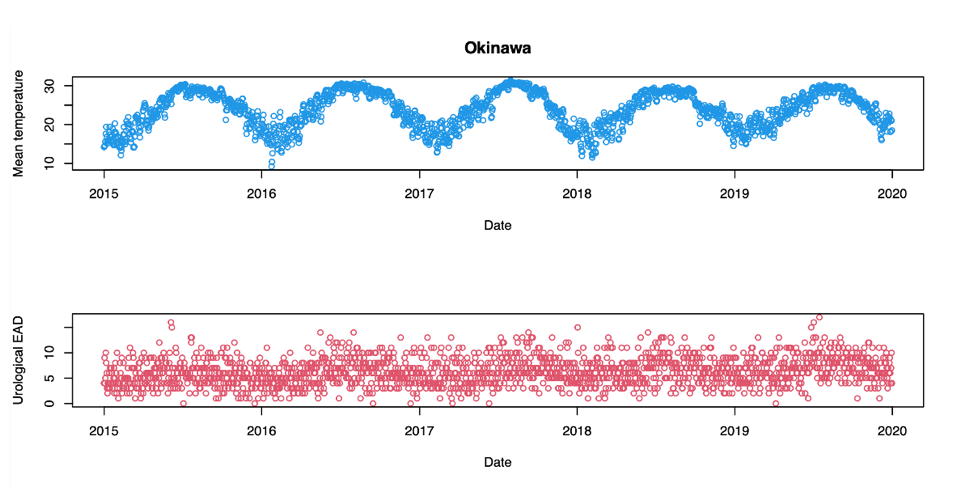  **Supplemental Figure 2.** Lag-response curve for heat effect on genitourinary emergency ambulance dispatches (EADs) at the country level in Japan for (A) overall population, as well as by (B) sex, (C) age and (D) disease severity sub-groups, with 95% empirical confidence interval (shaded).  **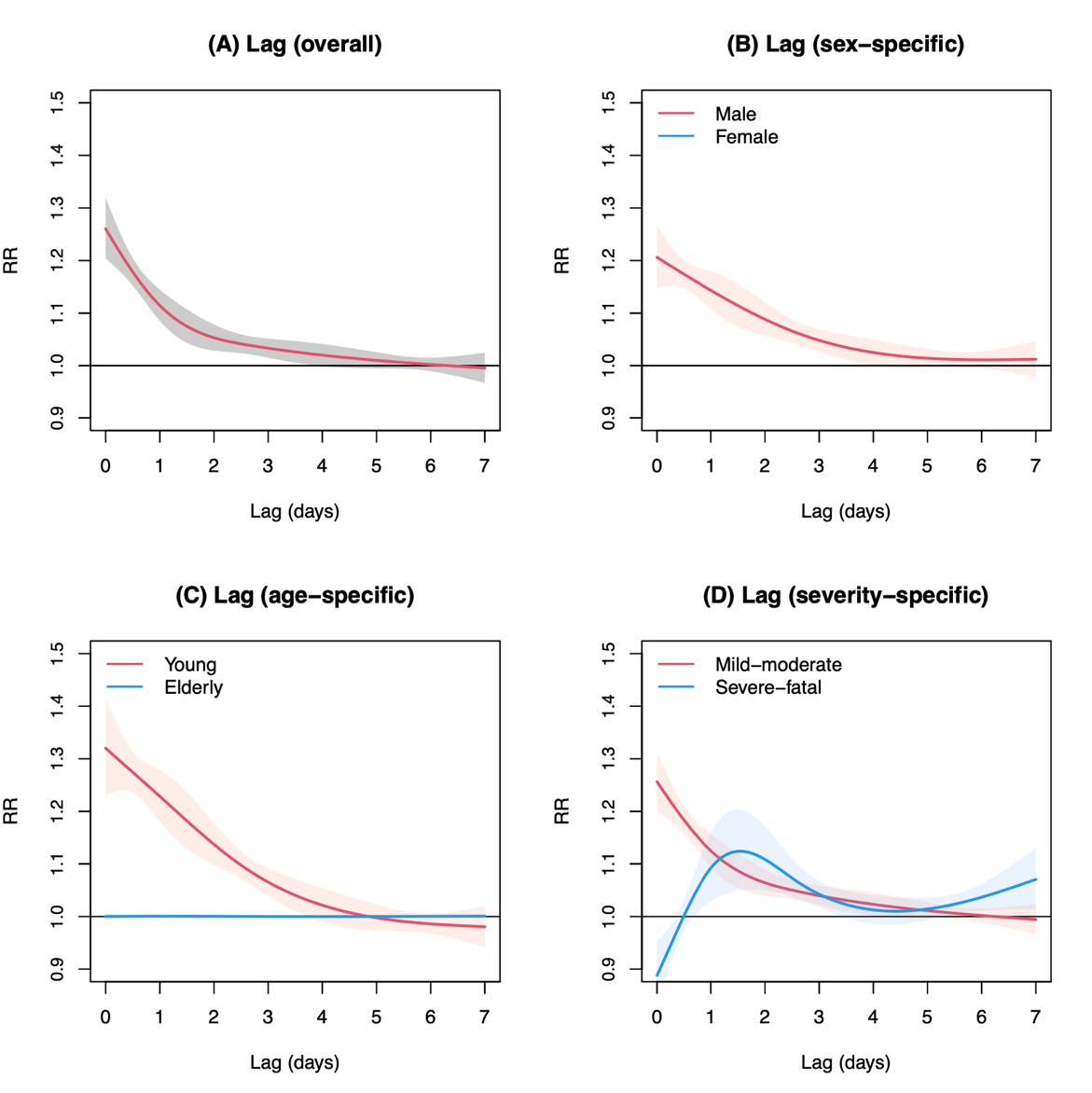**  **Supplemental Figure 3.** Exposure–response associations with and without best linear unbiased prediction (with 95% empirical CI, shaded grey) in representative prefectures of Japan. | |
| --- | --- |
| **Without BLUP** | **With BLUP** |
| 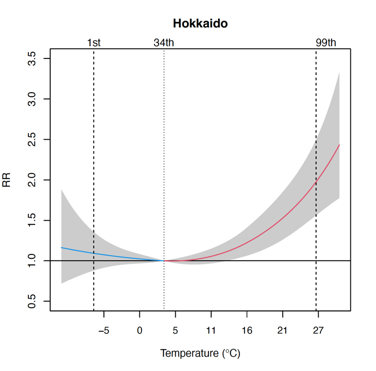 | 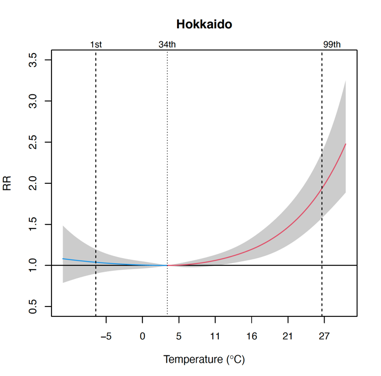 |
| 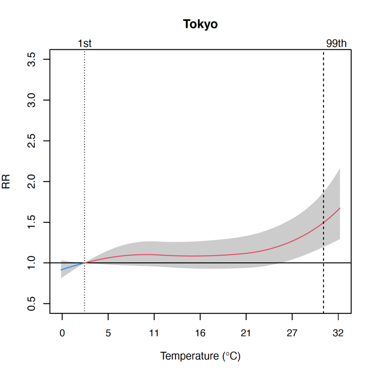 | 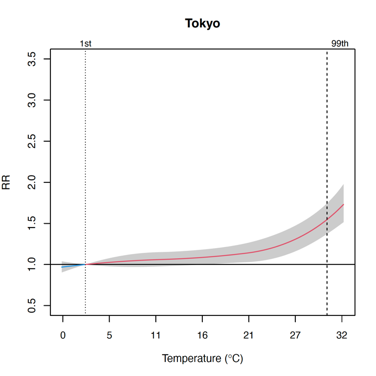 |
| 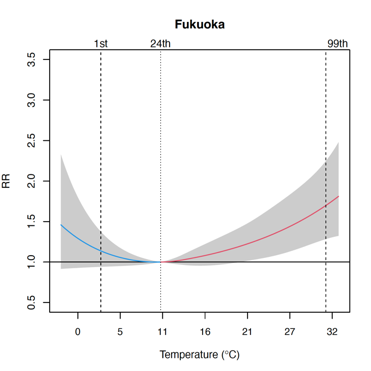 | 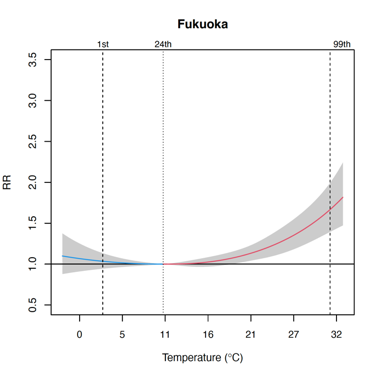 |
| 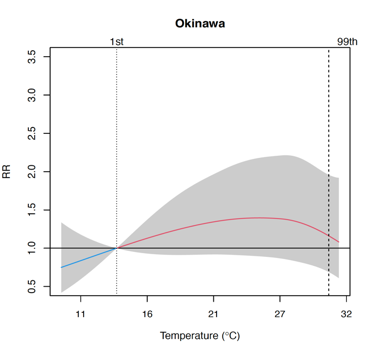 | 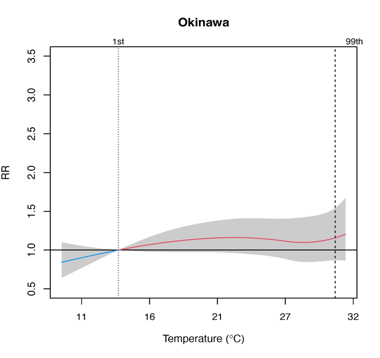 |

| **Supplemental Table 1.** Descriptive statistics for genitourinary emergency ambulance dispatches (EADs) from 2015 to 2019 in Japan^a^. | | |
| --- | --- | --- |
| **Prefecture** | **Total counts** | **Daily mean (SD)** |
| Hokkaido | 30,334 | 16.6 (4.7) |
| Aomori | 5,399 | 3.0 (1.8) |
| Iwate | 6,113 | 3.3 (1.8) |
| Miyagi | 8,681 | 4.8 (2.2) |
| Akita | 5,204 | 2.8 (1.8) |
| Yamagata | 5,747 | 3.1 (1.9) |
| Fukushima | 8,756 | 4.8 (2.2) |
| Ibaraki | 10,557 | 5.8 (2.6) |
| Tochigi | 6,596 | 3.6 (2.0) |
| Gunma | 8,032 | 4.4 (2.2) |
| Saitama | 29,701 | 16.3 (4.7) |
| Chiba | 26,073 | 14.3 (4.2) |
| Tokyo | 44,167 | 30.2 (6.8) |
| Kanagawa | 39,122 | 21.4 (5.5) |
| Niigata | 10,214 | 5.6 (2.6) |
| Toyama | 3,621 | 2.0 (1.4) |
| Ishikawa | 6,254 | 3.4 (2.0) |
| Fukui | 2,334 | 1.3 (1.2) |
| Yamanashi | 2,930 | 1.6 (1.3) |
| Nagano | 9,199 | 5.0 (2.4) |
| Gifu | 7,366 | 4.0 (2.1) |
| Shizuoka | 10,437 | 5.7 (2.7) |
| Aichi | 38,415 | 21.0 (5.5) |
| Mie | 16,090 | 8.8 (3.4) |
| Shiga | 5,190 | 2.8 (1.7) |
| Kyoto | 18,856 | 10.3 (3.6) |
| Osaka | 73,228 | 40.1 (10.6) |
| Hyogo | 29,264 | 16.0 (4.8) |
| Nara | 5,812 | 3.2 (1.9) |
| Wakayama | 6,057 | 3.3 (1.9) |
| Tottori | 1,461 | 0.8 (0.9) |
| Shimane | 3,240 | 1.8 (1.4) |
| Okayama | 11,918 | 6.5 (2.8) |
| Hiroshima | 10,394 | 5.7 (2.6) |
| Yamaguchi | 5,367 | 2.9 (1.8) |
| Tokushima | 4,697 | 2.6 (1.8) |
| Kagawa | 4,553 | 2.5 (1.7) |
| Ehime | 6,237 | 3.4 (2.0) |
| Kochi | 5,826 | 3.2 (1.9) |
| Fukuoka | 22,827 | 12.5 (4.0) |
| Saga | 3,572 | 2.0 (1.4) |
| Nagasaki | 5,066 | 2.8 (1.8) |
| Kumamoto | 11,211 | 6.1 (2.7) |
| Oita | 6,203 | 3.4 (1.9) |
| Miyazaki | 4,311 | 2.4 (1.5) |
| Kagoshima | 6,711 | 3.7 (2.0) |
| Okinawa | 11,563 | 6.3 (2.7) |

Note: SD, standard deviation.

^a^ For Tokyo, EAD data were available from 2016 to 2019.

| **Supplemental Table 2.** Summary statistics for meteorological variables from 2015 to 2019 at the prefecture level. | | | | | | | | |
| --- | --- | --- | --- | --- | --- | --- | --- | --- |
| **Prefecture** | **Meteorological variables (°C)** | **Mean** | **SD** | **Minimum** | **25th** | **Median** | **75th** | **Maximum** |
| Hokkaido | Mean temperature | 9.6 | 9.5 | -11.5 | 0.9 | 10.1 | 17.9 | 29.8 |
|  | Dew point | 3.6 | 10.1 | -110.1 | -4.9 | 2.8 | 12.2 | 23.3 |
| Aomori | Mean temperature | 11.2 | 8.9 | -7.4 | 2.7 | 11.7 | 19.0 | 30.0 |
|  | Dew point | 6.4 | 10.1 | -111.1 | -1.5 | 5.8 | 14.6 | 24.0 |
| Iwate | Mean temperature | 11.2 | 9.3 | -7.2 | 2.2 | 11.3 | 19.3 | 29.0 |
|  | Dew point | 6.2 | 12.0 | -112.0 | -2.0 | 6.1 | 15.3 | 24.3 |
| Miyagi | Mean temperature | 13.5 | 8.3 | -3.7 | 5.6 | 13.9 | 20.6 | 30.9 |
|  | Dew point | 7.8 | 9.5 | -10.0 | -1.0 | 7.2 | 16.5 | 25.5 |
| Akita | Mean temperature | 12.5 | 9.0 | -5.5 | 4.0 | 12.5 | 20.4 | 32.1 |
|  | Dew point | 7.5 | 10.7 | -111.8 | -0.6 | 7.3 | 15.6 | 25.1 |
| Yamagata | Mean temperature | 12.6 | 9.3 | -5.4 | 3.7 | 12.8 | 20.7 | 30.8 |
|  | Dew point | 7.5 | 9.3 | -110.3 | -0.7 | 6.6 | 15.6 | 24.3 |
| Fukushima | Mean temperature | 14.0 | 8.8 | -3.9 | 5.8 | 14.3 | 21.4 | 31.6 |
|  | Dew point | 8.1 | 11.2 | -111.7 | -0.2 | 7.8 | 16.9 | 25.1 |
| Ibaraki | Mean temperature | 14.8 | 8.2 | -0.8 | 7.1 | 15.3 | 21.5 | 30.7 |
|  | Dew point | 9.7 | 9.7 | -14.5 | 1.2 | 10.5 | 18.2 | 25.5 |
| Tochigi | Mean temperature | 14.9 | 8.5 | -1.9 | 6.8 | 15.5 | 21.9 | 30.9 |
|  | Dew point | 9.0 | 10.0 | -13.1 | 0.0 | 9.5 | 17.9 | 26.2 |
| Gunma | Mean temperature | 15.6 | 8.5 | -1.9 | 7.6 | 16.2 | 22.6 | 32.7 |
|  | Dew point | 8.0 | 11.1 | -112.0 | -1.1 | 8.1 | 17.3 | 25.5 |
| Saitama | Mean temperature | 16.0 | 8.4 | -0.7 | 8.2 | 16.5 | 23.0 | 33.5 |
|  | Dew point | 8.6 | 10.4 | -14.7 | -0.5 | 9.2 | 18.1 | 26.3 |
| Chiba | Mean temperature | 16.8 | 7.7 | 1.0 | 9.5 | 17.4 | 23.1 | 31.8 |
|  | Dew point | 10.0 | 10.2 | -16.0 | 1.5 | 11.3 | 18.8 | 25.9 |
| Tokyo | Mean temperature | 16.4 | 7.9 | 0.0 | 8.8 | 16.9 | 23.0 | 32.2 |
|  | Dew point | 10.2 | 11.4 | -111.2 | 1.5 | 11.5 | 19.3 | 26.7 |
| Kanagawa | Mean temperature | 16.8 | 7.6 | 0.7 | 9.6 | 17.4 | 22.8 | 31.6 |
|  | Dew point | 10.6 | 9.8 | -13.4 | 2.3 | 11.5 | 19.2 | 26.1 |
| Niigata | Mean temperature | 14.4 | 8.6 | -2.4 | 6.3 | 14.4 | 21.8 | 33.0 |
|  | Dew point | 9.4 | 9.9 | -111.8 | 1.7 | 8.8 | 17.3 | 25.7 |
| Toyama | Mean temperature | 15.0 | 8.7 | -2.9 | 7.0 | 15.4 | 22.3 | 32.2 |
|  | Dew point | 10.1 | 10.1 | -110.1 | 2.7 | 10.0 | 18.1 | 26.1 |
| Ishikawa | Mean temperature | 15.5 | 8.5 | -1.5 | 7.7 | 16.0 | 22.6 | 33.7 |
|  | Dew point | 9.6 | 8.5 | -6.3 | 1.9 | 9.1 | 17.7 | 24.8 |
| Fukui | Mean temperature | 15.3 | 8.8 | -1.9 | 7.3 | 15.8 | 22.7 | 32.4 |
|  | Dew point | 10.6 | 9.2 | -111.3 | 3.0 | 10.5 | 18.3 | 25.6 |
| Yamanashi | Mean temperature | 15.7 | 8.6 | -1.5 | 7.5 | 16.4 | 23.0 | 31.6 |
|  | Dew point | 7.9 | 10.4 | -19.6 | -0.7 | 9.0 | 16.8 | 24.8 |
| Nagano | Mean temperature | 12.8 | 9.4 | -5.9 | 3.8 | 13.5 | 21.1 | 30.9 |
|  | Dew point | 7.9 | 9.8 | -110.0 | -0.3 | 7.8 | 16.1 | 24.2 |
| Gifu | Mean temperature | 16.7 | 8.5 | -1.0 | 8.8 | 17.2 | 24.0 | 33.2 |
|  | Dew point | 9.8 | 9.2 | -11.5 | 1.5 | 9.8 | 18.0 | 25.2 |
| Shizuoka | Mean temperature | 17.4 | 7.4 | 1.7 | 10.7 | 18.1 | 23.5 | 31.2 |
|  | Dew point | 10.9 | 10.6 | -109.71 | 3.8 | 12.2 | 19.1 | 26.4 |
| Aichi | Mean temperature | 16.7 | 8.4 | -1.1 | 8.9 | 17.2 | 23.8 | 33.3 |
|  | Dew point | 9.5 | 10.2 | -110.0 | 1.1 | 10.1 | 18.2 | 25.6 |
| Mie | Mean temperature | 16.7 | 8.1 | 0.0 | 9.1 | 17.2 | 23.5 | 32.9 |
|  | Dew point | 9.8 | 9.2 | -15.5 | 1.3 | 10.1 | 18.1 | 26.0 |
| Shiga | Mean temperature | 15.6 | 8.5 | -1.4 | 7.6 | 15.9 | 22.7 | 31.7 |
|  | Dew point | 10.7 | 9.0 | -111.1 | 3.1 | 10.7 | 18.3 | 25.4 |
| Kyoto | Mean temperature | 16.8 | 8.6 | -0.6 | 8.5 | 17.3 | 24.1 | 32.6 |
|  | Dew point | 10.2 | 8.5 | -14.2 | 2.5 | 9.9 | 17.8 | 24.9 |
| Osaka | Mean temperature | 17.4 | 8.2 | 0.1 | 9.6 | 18.0 | 24.3 | 32.0 |
|  | Dew point | 10.6 | 8.9 | -13.7 | 2.7 | 10.8 | 18.7 | 25.0 |
| Hyogo | Mean temperature | 17.4 | 8.0 | -0.8 | 9.9 | 18.2 | 24.1 | 32.2 |
|  | Dew point | 10.6 | 9.9 | -111.4 | 2.7 | 11.0 | 18.8 | 25.8 |
| Nara | Mean temperature | 15.9 | 8.5 | -1.2 | 7.8 | 16.4 | 23.1 | 31.3 |
|  | Dew point | 10.7 | 9.6 | -112.0 | 3.1 | 11.1 | 18.8 | 24.9 |
| Wakayama | Mean temperature | 17.4 | 8.0 | -0.4 | 9.8 | 18.1 | 24.0 | 32.2 |
|  | Dew point | 10.9 | 11.0 | -111.1 | 3.2 | 11.6 | 19.3 | 25.9 |
| Tottori | Mean temperature | 15.7 | 8.4 | -3.5 | 8.0 | 16.2 | 22.7 | 32.2 |
|  | Dew point | 10.6 | 10.5 | -110.8 | 3.3 | 10.5 | 18.5 | 25.5 |
| Shimane | Mean temperature | 15.7 | 8.2 | -3.5 | 8.1 | 16.2 | 22.4 | 32.3 |
|  | Dew point | 11.3 | 9.2 | -110.6 | 4.0 | 11.4 | 18.9 | 26.0 |
| Okayama | Mean temperature | 16.3 | 8.5 | -2.2 | 8.1 | 16.6 | 23.4 | 32.1 |
|  | Dew point | 10.0 | 13.3 | -112.0 | 2.7 | 10.9 | 18.7 | 25.5 |
| Hiroshima | Mean temperature | 16.9 | 8.3 | -2.2 | 9.1 | 17.3 | 23.7 | 32.8 |
|  | Dew point | 9.6 | 8.7 | -9.4 | 1.8 | 9.5 | 17.5 | 24.6 |
| Yamaguchi | Mean temperature | 16.1 | 8.4 | -4.2 | 8.3 | 16.6 | 23.2 | 31.2 |
|  | Dew point | 11.4 | 10.6 | -111.6 | 4.2 | 11.8 | 19.1 | 26.2 |
| Tokushima | Mean temperature | 17.2 | 7.8 | -2.1 | 9.7 | 17.8 | 23.6 | 32.5 |
|  | Dew point | 11.4 | 9.1 | -9.4 | 3.7 | 11.8 | 19.5 | 26.2 |
| Kagawa | Mean temperature | 17.1 | 8.2 | -1.2 | 9.2 | 17.5 | 24.0 | 32.6 |
|  | Dew point | 10.9 | 9.4 | -112.0 | 3.2 | 11.1 | 19.1 | 25.5 |
| Ehime | Mean temperature | 17.2 | 7.9 | -0.7 | 9.8 | 17.6 | 23.6 | 31.8 |
|  | Dew point | 11.0 | 9.6 | -109.5 | 3.6 | 11.4 | 19.1 | 25.4 |
| Kochi | Mean temperature | 17.6 | 7.6 | -0.2 | 10.7 | 18.4 | 24.0 | 31.6 |
|  | Dew point | 11.7 | 11.2 | -109.8 | 4.1 | 12.6 | 20.3 | 26.2 |
| Fukuoka | Mean temperature | 17.8 | 7.7 | -2.0 | 10.7 | 18.1 | 24.0 | 32.8 |
|  | Dew point | 12.0 | 9.2 | -110.2 | 4.6 | 12.6 | 19.9 | 26.6 |
| Saga | Mean temperature | 17.5 | 8.1 | -3.3 | 10.0 | 18.0 | 24.2 | 32.5 |
|  | Dew point | 12.0 | 8.6 | -9.3 | 4.7 | 12.3 | 19.6 | 26.4 |
| Nagasaki | Mean temperature | 17.7 | 7.5 | -2.4 | 10.8 | 18.3 | 23.7 | 32.4 |
|  | Dew point | 12.7 | 9.9 | -110.2 | 5.7 | 13.4 | 20.5 | 27.1 |
| Kumamoto | Mean temperature | 17.5 | 8.2 | -3.0 | 10.0 | 18.3 | 24.3 | 31.9 |
|  | Dew point | 12.1 | 10.3 | -109.9 | 5.0 | 12.7 | 20.2 | 26.1 |
| Oita | Mean temperature | 17.2 | 7.7 | -1.7 | 10.0 | 17.7 | 23.3 | 31.3 |
|  | Dew point | 11.7 | 9.7 | -110.0 | 4.5 | 12.2 | 19.7 | 26.2 |
| Miyazaki | Mean temperature | 18.1 | 7.3 | 0.3 | 11.5 | 18.7 | 24.2 | 31.4 |
|  | Dew point | 13.3 | 11.1 | -109.5 | 7.1 | 14.5 | 21.3 | 26.9 |
| Kagoshima | Mean temperature | 19.1 | 7.3 | -0.4 | 12.6 | 19.8 | 25.3 | 31.4 |
|  | Dew point | 13.9 | 9.8 | -110.8 | 7.3 | 14.9 | 21.6 | 27.7 |
| Okinawa | Mean temperature | 23.8 | 4.7 | 9.2 | 20.1 | 24.1 | 28.2 | 31.4 |
|  | Dew point | 18.7 | 6.3 | 1.0 | 14.2 | 19.8 | 24.5 | 27.2 |

Note: SD, standard deviation; 25th and 75th are percentiles.

| **Supplemental Table 3.** Multivariate Wald test on significance of each meta-predictor in explaining variation in overall cumulative temperature-mortality association in the second stage mixed-effects meta-regression models. Cochran Q-test for heterogeneity, I2 statistics for residual heterogeneity, Akaike information criteria (AIC) for model fitness. The last model selected by forward stepwise procedure includes both meta-predictors. |
| --- |

| Model | Predictor | Test for predictor  (*p*-value) | Q test  (*p*-value) | *I^2^* | AIC |
| --- | --- | --- | --- | --- | --- |
| Model 1 | Prefecture  (random=~1\|pref) | — | 0.030 | 17.0% | 107.1128 |
| Model2 | +Average temperature | 0.0611 | 0.082 | 13.0% | 105.9530 |
| Model 3 | +Temperature range | 0.2944 | 0.075 | 13.4% | 105.5388 |
| Model 4 | Full Model | 0.2318 | 0.149 | 10.0% | 102.8375 |
| Stepwise-selected model | | Model 4 (+Average temperature + Temperature range) | | | |

| **Supplemental Table 4.** Prefecture-specific association between temperature and genitourinary emergency ambulance dispatches (EADs) in Japan among the overall population. | | | | | |
| --- | --- | --- | --- | --- | --- |
| **Prefecture** | **MMTP** | | **MMT (^o^C)** | **RR_heat_ (95% CI)** | **RR_cold_ (95% CI)** |
| Hokkaido | | 1 | -6.7 | 2.0 (1.4, 2.8) | 1.00 |
| Aomori | | 1 | -4.3 | 2.5 (1.1, 5.7) | 1.00 |
| Iwate | | 1 | -4.3 | 1.5 (0.7, 3.3) | 1.00 |
| Miyagi | | 1 | -0.9 | 2.6 (1.3, 5.1) | 1.00 |
| Akita | | 1 | -2.5 | 0.9 (0.4, 2.2) | 1.00 |
| Yamagata | | 1 | -2.9 | 1.1 (0.5, 2.6) | 1.00 |
| Fukushima | | 1 | -0.8 | 2.8 (1.5, 5.1) | 1.00 |
| Ibaraki | | 1 | 1.2 | 1.3 (0.7, 2.5) | 1.00 |
| Tochigi | | 1 | 0.4 | 1.6 (0.8, 3.5) | 1.00 |
| Gunma | | 1 | 1.2 | 2.4 (1.4, 4.1) | 1.00 |
| Saitama | | 1 | 1.6 | 2.1 (1.5, 2.9) | 1.00 |
| Chiba | | 1 | 3.3 | 1.6 (1.1, 2.3) | 1.00 |
| Tokyo | | 1 | 2.7 | 1.5 (1.2, 2.0) | 1.00 |
| Kanagawa | | 1 | 3.3 | 1.6 (1.2, 2.2) | 1.00 |
| Niigata | | 12 | 3.4 | 1.7 (1.0, 2.9) | 1.0 (0.9, 1.2) |
| Toyama | | 10 | 3.1 | 1.4 (0.6, 3.0) | 1.0 (0.9,1.1) |
| Ishikawa | | 16 | 5.5 | 2.6 (1.4, 5.0) | 1.0 (0.8, 1.2) |
| Fukui | | 9 | 3.2 | 1.0 (0.3, 3.1) | 1.0 (0.9, 1.1) |
| Yamanashi | | 1 | 0.3 | 2.5 (0.9, 7.3) | 1.00 |
| Nagano | | 1 | -3.1 | 1.4 (0.7, 2.5) | 1.00 |
| Gifu | | 1 | 0.9 | 1.7 (0.9, 3.4) | 1.00 |
| Shizuoka | | 1 | 4.1 | 1.3 (0.7, 2.3) | 1.00 |
| Aichi | | 1 | 1.6 | 1.5 (1.2, 2.0) | 1.00 |
| Mie | | 1 | 2.6 | 1.9 (1.2, 3.1) | 1.00 |
| Shiga | | 1 | 0.8 | 1.5 (0.6, 3.6) | 1.00 |
| Kyoto | | 1 | 1.6 | 1.6 (1.0, 2.6) | 1.00 |
| Osaka | | 1 | 3.0 | 2.3 (1.7, 3.2) | 1.00 |
| Hyogo | | 1 | 2.5 | 1.3 (0.9, 1.9) | 1.00 |
| Nara | | 1 | 1.2 | 2.6 (1.1, 6.0) | 1.00 |
| Wakayama | | 1 | 3.1 | 3.0 (1.5, 6.0) | 1.00 |
| Tottori | | 1 | 0.4 | 3.4 (0.7, 15.9) | 1.00 |
| Shimane | | 6 | 4.0 | 2.1 (0.8, 5.3) | 1.0 (0.9, 1.1) |
| Okayama | | 23 | 7.6 | 2.1 (1.4, 3.2) | 1.0 (0.9, 1.2) |
| Hiroshima | | 1 | 1.7 | 2.0 (1.1, 3.6) | 1.00 |
| Yamaguchi | | 24 | 8.0 | 1.9 (1.0, 3.6) | 1.0 (0.9, 1.2) |
| Tokushima | | 27 | 10.2 | 1.7 (0.8, 3.4) | 1.0 (0.9, 1.2) |
| Kagawa | | 16 | 7.6 | 2.0 (1.0, 4.0) | 1.0 (0.9, 1.2) |
| Ehime | | 1 | 2.5 | 1.3 (0.6, 2.7) | 1.00 |
| Kochi | | 1 | 2.7 | 2.4 (1.2, 4.9) | 1.00 |
| Fukuoka | | 18 | 9.2 | 1.6 (1.2, 2.2) | 1.0 (0.9, 1.2) |
| Saga | | 33 | 12.7 | 2.2 (1.2, 4.0) | 1.0 (0.9, 1.2) |
| Nagasaki | | 30 | 12.4 | 2.1 (1.2, 3.8) | 1.0 (0.9, 1.2) |
| Kumamoto | | 1 | 1.7 | 1.5 (0.9, 2.4) | 1.00 |
| Oita | | 1 | 3.0 | 2.0 (1.0, 4.0) | 1.00 |
| Miyazaki | | 1 | 3.9 | 1.5 (0.7, 3.3) | 1.00 |
| Kagoshima | | 1 | 4.0 | 1.3 (0.6, 2.6) | 1.00 |
| Okinawa | | 1 | 13.6 | 1.2 (0.7, 2.1) | 1.00 |

Note: MMTP, minimum risk temperature percentile; MMT, minimum risk temperature; RR, relative risk; CI, confidence interval.

| **Supplemental Table 5.** Pooled RRs of genitourinary emergency ambulance dispatch (EAD) in Japan without adjustment for dew point. | | | | | |
| --- | --- | --- | --- | --- | --- |
|  | **Temperature percentile^a^** | | | **RRs (95% CI)** | |
|  |  | **MMTP^b^ (MMT)** |  | **Cold risk^c^** | **Heat risk^c^** |
| **Overall** |  | 1 (-1.4) |  | 1.00 | 1.61 (1.50, 1.73) |
| **Sex** |  |  |  |  |  |
| Male |  | 18 (6.7) |  | 1.03 (0.96, 1.11) | 1.66 (1.55, 1.77) |
| Female |  | 1 (-1.4) |  | 1.00 | 1.62 (1.47, 1.80) |
| **Age category (year)** |  |  |  |  |  |
| Younger (< 65) |  | 1 (-1.4) |  | 1.00 | 1.98 (1.77, 2.21) |
| Older (≥ 65) |  | 1 (-1.4) |  | 1.00 | 1.32 (1.20, 1.46) |
| **Severity category** |  |  |  |  |  |
| Mild or moderate |  | 1 (-1.4) |  | 1.00 | 1.64 (1.52, 1.76) |
| Severe or life-threatening |  | 83 (25.1) |  | 1.00 | 1.04 (0.87, 1.24) |
| Note: Pooled cumulative RRs with 95% CIs for genitourinary EADs for overall and subpopulations, estimated by using a conditional Poisson model adjusting for seasonality, long-term time trend, day of week and holiday. CI, confidence interval; MMTP, minimum risk temperature percentile; RR, risk ratio.  ^a^ The percentiles of the temperature for centering and RR calculation.  ^b^ The percentile of minimum risk temperature, identified between the 1st and 99th percentiles of temperature in country level.  ^c^ The cold and heat risks are the RRs at the 1^st^ (cold) and 99^th^ (heat) percentiles of temperature. | | | | | |

**Supplemental Table 6.** Alternative statistical modelling for the pooled cumulative and lag associations between temperature and genitourinary emergency ambulance dispatch (EAD) at the country level.


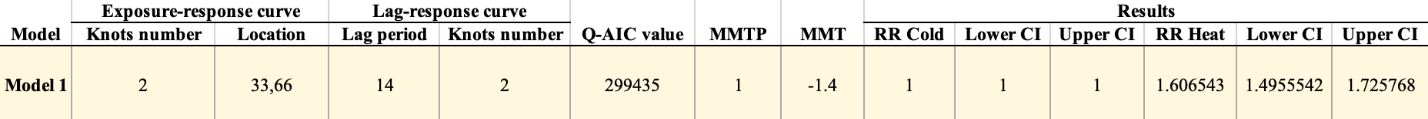


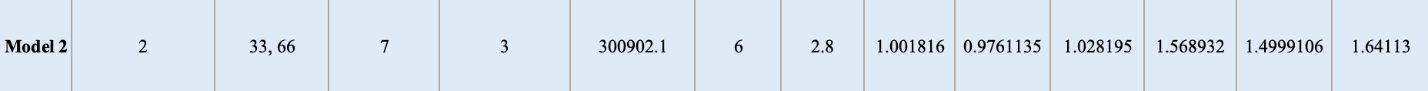


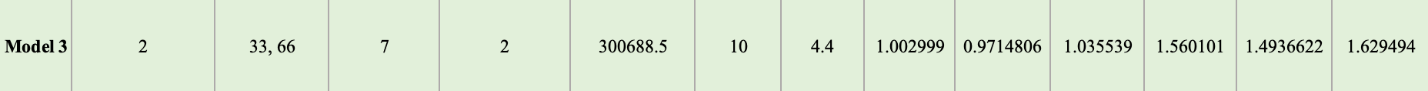


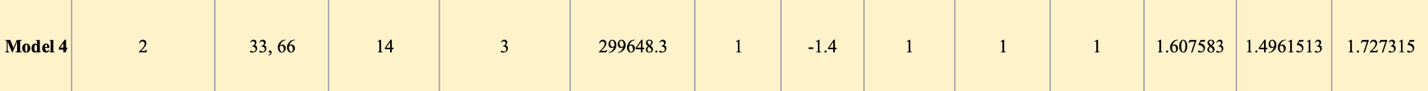


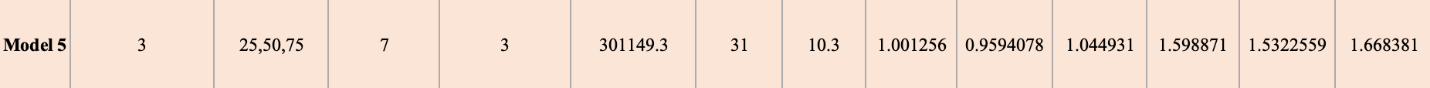


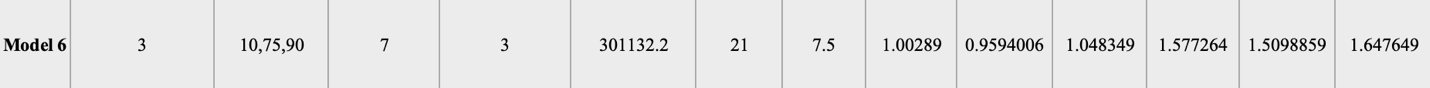


Note: Q-AIC, quasi-Akaike information criterion; MMTP, minimum risk temperature percentile; MMT, minimum risk temperature; RR, relative risk; CI, confidence interval.
